# Proximal venous ultrasound with risk stratification safely excludes deep venous thrombosis in emergency department routine care: an observational study

**DOI:** 10.1186/s13049-025-01382-7

**Published:** 2025-05-14

**Authors:** Maroan Cherkaoui, Mohammed Al-Attabi, Sara Salimi, Bader Cherkaoui, Jakob L Forberg

**Affiliations:** 1 Departmenf of Emergency Medicine, Helsingborg Hospital, Helsingborg, Sweden; 2 Department of Clinical Sciences, Lund University, Lund, Sweden

**Keywords:** Proximal compression ultrasound, LCU, Limited compression ultrasound, Deep vein thrombosis, DVT, Emergency department, ED, POCUS, Point-of-care ultrasound.

## Abstract

**Background:**

Lower limb deep vein thrombosis (DVT) is common in emergency departments (EDs) and can be fatal if left untreated due to the risk of progression to pulmonary embolism (PE). In Scandinavia, DVT diagnosis typically relies on ultrasound performed outside the ED in the diagnostic departments. However, international guidelines now recommend combining limited/proximal compression ultrasound of the lower extremity PUL with risk stratification as a viable approach for diagnosing and ruling out DVT. The aim of this study was to evaluate the safety of ruling out DVT by integrating PUL with risk stratification in ED routine care.

**Methods:**

This observational cohort study was conducted at the Helsingborg Hospital ED, Sweden, from April 2022 to November 2024. Adult patients with suspected DVT underwent PUL combined with risk stratification using the Wells score. Risk stratification, PUL findings, diagnosis and management plan were prospectively recorded. A 30-day follow-up was conducted to identify any subsequent DVT, PE or deaths registered as caused by PE post index visit. Patients prescribed anticoagulation following an ED-diagnosed DVT were followed up at 3 and 6 months to monitor for major bleeding events.

**Results:**

A total of 560 patients were evaluated, with an overall DVT prevalence of 18.4%. Of these, 471 patients (82.5%) were managed entirely within the ED, without referral to the diagnostic department. Of the 381 patients discharged from the ED with DVT ruled out (negative PUL and low risk assessment), two were diagnosed with DVT or PE within 30 days. This resulted in a negative predictive value of 99.5% (95% CI: 98–99.9%) and a sensitivity of 97.8% (95% CI: 92.4–99.7%) for PUL combined with low-risk stratification in ruling out DVT. One of the 90 patients diagnosed with DVT in the ED and prescribed anticoagulant therapy experienced a major bleed related to an in-hospital procedure.

**Conclusions:**

In this single-center ED study the combination of PUL and risk stratification in routine care was a safe and effective method for the early diagnosis and ruling out DVT. Using this approach, more than 8 out of 10 patients could be diagnosed in the ED without the need for external diagnostic support.

## Background

Lower limb deep-vein thrombosis (DVT) is common in emergency departments (ED) and can be fatal if untreated. Prompt and accurate ED diagnosis is crucial because missed DVT can cause morbidity and mortality. Untreated, a third of DVT cases can progress to pulmonary embolism (PE), which has a short-term mortality rate exceeding 20% [1, 2]. The diagnosis of DVT in Scandinavia has traditionally been performed by diagnostic specialists, such as radiologists and sonographers, who conduct the examination in a diagnostic department outside the ED during office hours. Despite its lengthy procedure time, duplex color-flow ultrasound (CDUS) remains the standard in many Scandinavian hospitals. National and international guidelines have replaced CDUS with whole-leg compression ultrasound (WUL) or limited/proximal compression ultrasound of the lower extremity (PUL) [3, 4] due to the disadvantage of CDUS having longer training requirements and a longer exam time. When using the simpler and less time-consuming PUL, it must be combined with risk stratification, as high-risk patients with a negative PUL require either a negative d-dimer or a repeat scan to rule out DVT [4]. Since examinations through a diagnostic department are often restricted to office hours, patients have traditionally been bridged with anticoagulation therapy until a scheduled examination, often, the following weekday [3, 5]. The recent adoption of point-of-care ultrasound (POCUS) in Scandinavian EDs now allows for on-site DVT diagnosis and treatment, even outside office hours. This development offers potential benefits for both patients and ED logistics. Performing PUL in the ED could reduce the need for referral to the diagnostic department, decrease patient length of stay, and limit unnecessary anticoagulation therapy. Prior studies using POCUS to diagnose DVT in the ED have demonstrated that PUL has high diagnostic accuracy with a sensitivity of 90-96% and a specificity of 95-98.5% [6–9]. These studies often demonstrate that higher diagnostic accuracy requires emergency physicians (EPs) who are specialists rather than residents or who have undergone considerable ultrasound training [8, 10]. However, if PUL is to be implemented in routine 24/7 care, emergency medicine (EM) physicians with varying levels of experience and limited PUL training will need to be able to perform the examinations safely. Apart from a small pilot study [11] to our knowledge, there are no Scandinavian studies evaluating PUL in the ED, and few studies have examined its use in routine care when combined with risk stratification and performed by EM physicians with varying levels of experience.

The aim of this study was to evaluate the safety of ruling out DVT by integrating PUL with risk stratification in routine ED care, performed by EM physicians with diverse training and ultrasound experience.

## Methods

### Setting

This observational cohort study was conducted at the ED of Helsingborg Hospital, a community-based teaching hospital in Sweden. The ED has an annual census of approximately 80,000 patient visits. The study period was from April 2022 until November 2023.

### Ultrasound training and examination

All PUL examinations were performed using a 9 L-RS (2.4–10.5 MHz) GE probe on a GE Venue R1 machine. Emergency medicine physicians and residents received ultrasound training, which included an 8-hour didactic and practical session, along with a minimum of 20 PUL examinations to complete the course. Furthermore, PUL certification required passing a multiple-choice test and a direct observation of procedural skills assessment. According to the training protocol PUL involved compression testing for thrombus every centimeter, from the inguinal ligament (end of the external iliac vein) to the trifurcation of the popliteal vein into anterior tibial vein, posterior tibial vein and fibular vein. If the trifurcation could not be identified, the distal end of the exam was defined as 5 cm distal to the knee crease. Additionally, scanning the symptomatic area was a requirement, particularly if the patient had symptoms below the popliteal vein, such as pain.

### Patient selection

We included adult patients (≥ 18 years of age) presenting to the ED with signs and symptoms suggestive of DVT, using a convenience sampling method based on the availability of a PUL certified emergency physician or resident. Patients were included if they underwent a PUL performed by a certified specialist or resident, with the results of the exam and risk stratification criteria documented prospectively on a dedicated datasheet. Patients were excluded if they were not residents of Sweden, as this would prevent follow-up.

### Examiner characteristics

A total of 10 examiners were certified to perform PUL at the beginning of the study; 4 were emergency medicine specialists, and 6 were residents. An additional six examiners were certified during the data collection period. The median number of PUL exams per examiner at the start of the study was 49, with a minimum of 34 and a maximum of 98.

### Diagnostic algorithm

According to regional guidelines a negative PUL combined with low risk stratification ruled out DVT. Low risk was defined as having any one of the following criteria:

1. Clinically unlikely DVT (Wells score ≤ 1 point).
2. Negative D-dimer test, applicable if symptom onset within ≤ 1 week.
3. Identification of another lesion that better explains the presenting symptoms than DVT.

**Fig. 1:**
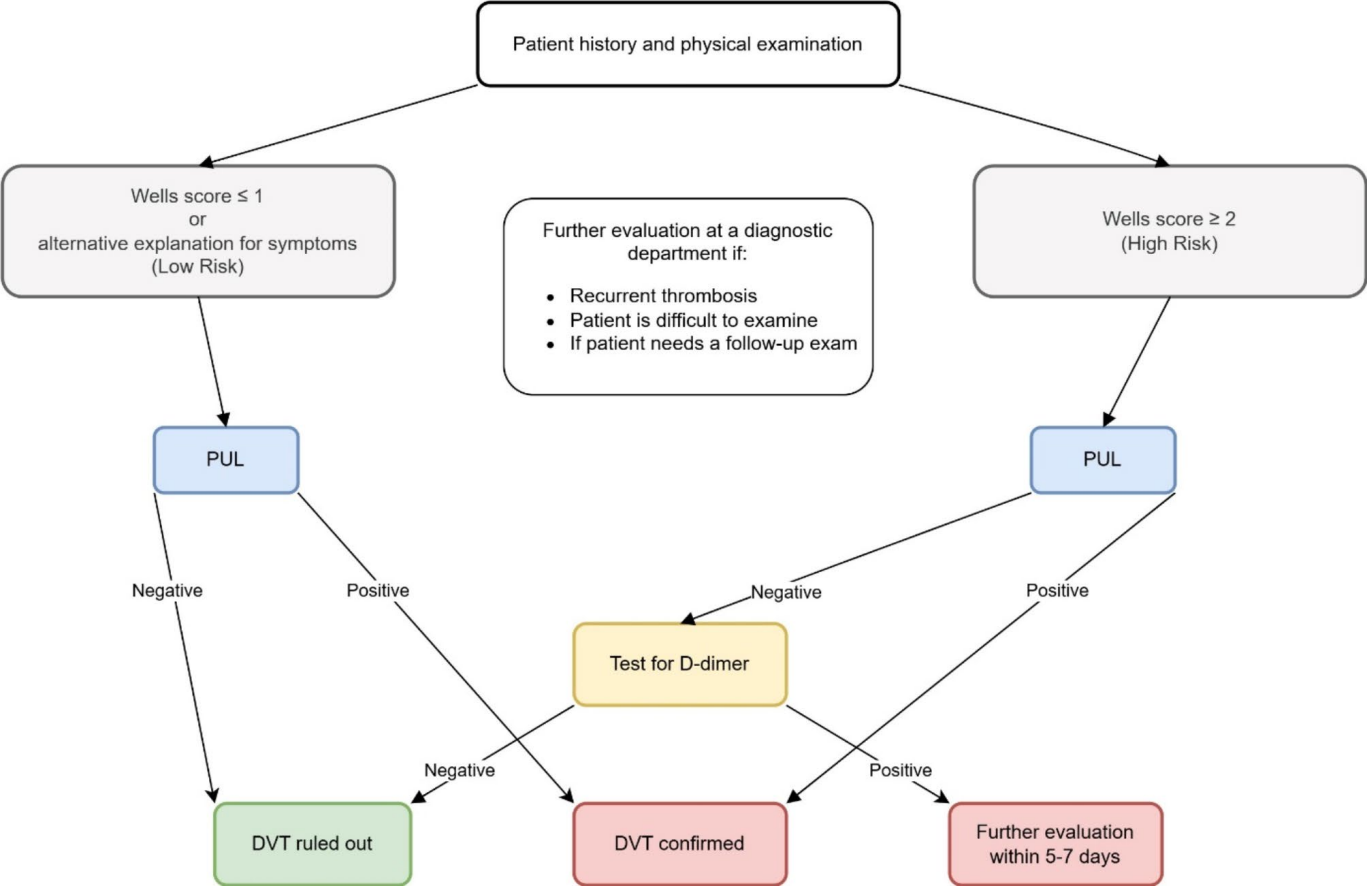
Management algorithm for suspected deep venous thrombosis using proximal compression ultrasound. PUL: proximal compression ultrasound, DVT: deep venous thrombosis

Patients with a negative PUL who met any of these criteria of low risk had DVT ruled out and were managed solely within the ED without further imaging. If a DVT could not be ruled out, the patients were referred to the diagnostic department, where CDUS was performed during office hours for definitive evaluation (Fig. 1).

A 30-day follow-up was conducted to determine whether any patients who were ruled out for DVT in the ED, without external diagnostic support, subsequently developed a DVT or PE. A true positive diagnosis was defined as one of the following: a final diagnosis of DVT established in the emergency department or diagnostic department; a diagnosis of DVT or pulmonary embolism (PE) within 30 days of follow up; or a death registered as caused by PE within the same time frame.

An additional follow up at 3 and 6 months (the recommended anticoagulation treatment duration) was conducted to identify any bleeding complications in patients diagnosed with DVT and started on anticoagulation therapy in the ED, using the International Society on Thrombosis and Haemostasis definition of major and non-major bleeding [12].

In collaboration with biostatisticians, power calculations were performed. Assuming a negative predictive value (NPV) of 99% and a desired precision of ± 1%, a total of 381 negative PUL examinations were required.

### Data collection

For each included patient, risk stratification criteria, diagnosis, and management plan were prospectively recorded on a datasheet during the index visit. This documentation encompassed all information required for calculating the Wells score, the PUL results, as well as the diagnosis and management, including referrals to the diagnostic department. Follow-up data were collected from patient records within 30 days, including a review of electronic records from all hospitals and outpatient diagnostic departments in the region.

### Statistical analysis

Patient and ultrasound examiner characteristics were reported using descriptive statistics. Normally distributed continuous data were reported as means with standard deviations (SD), and non-normally distributed data as medians with 25–75% interquartile ranges (IQRs). Data analysis was conducted using SPSS for Windows, version 23. Sensitivity, NPV and prevalence are presented with 95% confidence intervals calculated using the MedCalc diagnostic test evaluation calculator [13].

### Ethics

In accordance with Swedish law, the study was approved by a Swedish ethics review board (Dnr 2023-05183-01) and by Region Skåne.

## Results

**Fig. 2:**
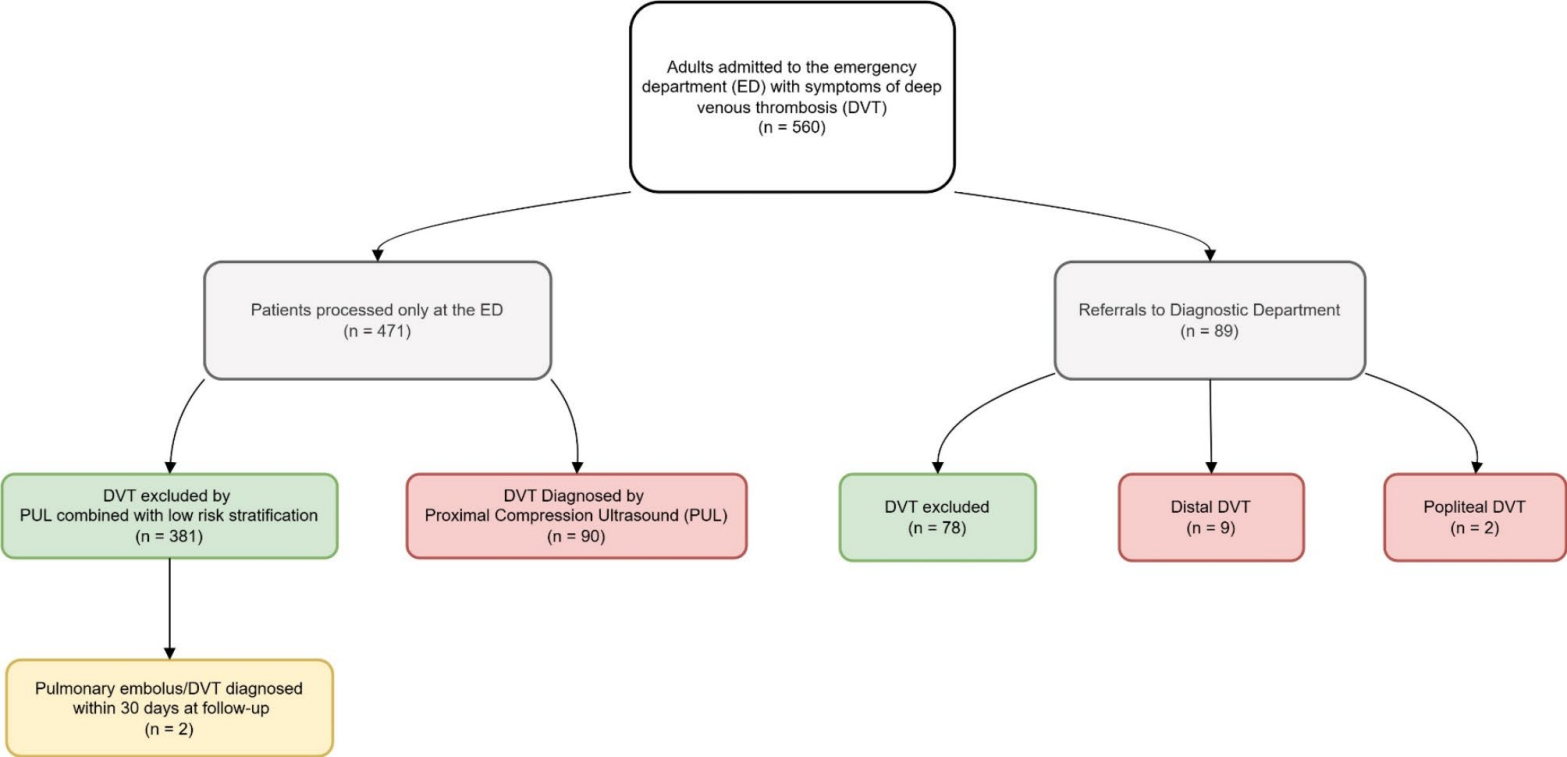
Flowchart of patient inclusion, disposition and diagnosis. PUL: proximal compression ultrasound, DVT: deep venous thrombosis. ED: emergency department

A total of 561 patients with suspected DVT were evaluated using PUL, with documented risk stratification and corresponding management (Fig. 2). Of these, 472 patients (82.5%) were managed entirely at the ED without the need for referral to a diagnostic department (ED group). The overall prevalence of DVT among all included patients was 18.4% (95% CI: 15.3-21.9%), with a prevalence of 19.5% (95% CI: 16-23.4%) observed in the ED group and 12.4% (95% CI: 6.3-21%) in patients referred to a diagnostic department. In the ED group, DVT was excluded in 381 patients, while 90 were diagnosed with DVT. Among the 89 patients referred to the diagnostic department, 88 initially had a negative PUL in the ED. The diagnostic department detected DVT in 11 of the 89 referred patients (9 distal DVTs and 2 located in the popliteal vein). One patient was referred to the diagnostic department despite having a positive PUL, due to a concern that chronic changes might be mistaken for a recurrent thrombosis and was ultimately not diagnosed with DVT. One patient, aged over 90 years, who was ruled out for DVT in the ED and had advanced terminal-phase lung cancer, died during the follow-up period. The death certificate did not list PE as the cause of death.

In two patients (0.5%), DVT and PE were diagnosed within 30 days, despite initially being ruled out for DVT in the ED. The negative predictive value and sensitivity of PUL combined with risk stratification for ruling out DVT were 99.5% (95% CI: 98-99.9%) and 97.8% (95% CI: 92.4-99.7%), respectively.

Two patients were referred to the diagnostic department due to high risk and were diagnosed with proximal DVT, despite it not being detected on the initial PUL in the ED. Therefore, if risk stratifications had been omitted, the NPV and sensitivity of ruling out proximal DVT using only PUL in the ED would have been 99.2% (95% CI: 97.8-99.7%) and 95.7% (95% CI: 89.5-98.8%), respectively. Nine patients, who initially had a normal PUL, were diagnosed with distal DVT in the calf after being referred to the diagnostic department due to a high-risk estimate. A total of 11 cases of DVT (2%) would have been missed if PUL had been used alone, without referring high risk patients. The overall NPV and sensitivity of PUL alone would have been 97.2% (95% CI: 95.5-98.3%) and 87.4% (95% CI: 79.4-93.1%) of diagnosing both proximal and distal DVT. Reporting specificity would not be meaningful, as false positives were not systematically evaluated in the study.

Of the two cases where DVT was initially ruled out in the ED but had a positive outcome within 30 days (false negatives), the first patient presented to the ED four weeks post knee surgery with pain localized to the medial joint space of the knee. The initial Wells score was 3, but after the treating physician deducted 2 points for the more likely alternative diagnosis of symptoms related to recent knee surgery, the patient was classified as low risk for DVT. The initial PUL was negative, and it was documented, according to department protocol, that no sonographic findings of veins were seen at the symptomatic area. When the patient returned after one week due to dyspnea, a computed tomography angiography revealed a PE. The second patient was receiving prophylactic dosing of low molecular weight heparin (LMWH) due to cast immobilization after an Achillies tendon rupture. Seven days post-injury, the patient presented with pain at the Achilles tendon. The initial PUL was negative, and the patient was assigned 1 point on the Wells score for cast immobilization. The patient returned to the ED 20 days later with a new complaint of calf pain that had persisted for the past few days only. A subsequent CDUS by the diagnostic department confirmed a distal DVT in the fibular vein.

A follow-up at 90 and 180 days was conducted for 101 patients treated with anticoagulation following a diagnosed DVT. Among these, 1 patient (0.99%) experienced a major bleed, while 4 patients (3.96%) had non-major bleeding events. Only one major bleed occurred, during follow-up, in a patient with DVT diagnosed in the ED, where direct oral anticoagulation therapy was initiated. This patient returned to the hospital with vomiting and abdominal pain (initially without signs of bleeding) and was subsequently diagnosed with biliary obstruction. Following endoscopic intervention, the patient’s hemoglobin levels dropped significantly, raising suspicion of an upper gastrointestinal bleed. Given the patient’s frailty, confirmatory gastroscopy was not performed, however, the bleeding resolved upon cessation of anticoagulation therapy. The non major bleeding events included hematuria related to bladder cancer, hemoptysis in a patient with sarcoidosis, menorrhagia and a gastrointestinal bleed due to an ulcerative colitis flare-up.

### Patient characteristics

**Table 1:** Patient characteristics. DVT, deep venous thrombosis; PE, pulmonary embolus; IQR, interquartile range

	Diagnosed at the Emergency Department			Diagnosed at the Diagnostic department	
	DVT ruled-out		DVT ruled-in	DVT ruled-out	DVT ruled-in
Age (IQR 25–75)	62 (48–76)		70 (56–78)	68 (52–76)	54 (49–71)
Female	222 (58%)		39 (42%)	42 (54%)	4 (36%)
Previous DVT or PE	64 (17%)		13 (14%)	16 (21%)	1 (9,1%)
Active cancer	12 (3%)		12 (13%)	8 (10%)	0 (0%)
Wells score > 1	48 (13%)		83% (90%)	68 (87%)	10 (91%)
Median duration of symptoms in days (IQR 25–75)	4 (2-9.5)		4 (2–10)	6 (3–14)	7 (3–21)

**Table 2:** Most common final diagnoses of patients where deep vein thrombosis was ruled out

Final Diagnosis	Number of cases
Muscle pain	148 (39%)
Leg oedema	56 (15%)
Thromboflebitis	43 (11%)
Bakercyst	37 (10%)
Erysipelas	32 (8.4%)
Knee joint pathology	20 (5.2%)
Leg injury	12 (3.1%)
Varicose vein	10 (2.6%)
Ankle joint pain or swelling	8 (2.1%)
Hematoma (including intramuscular)	6 (1.6%)

## Discussion

This study demonstrates that combining PUL with risk stratification is a safe and effective method for ruling out DVT in the ED when integrated into routine care. We showed that PUL, combined with a risk stratification algorithm including the Wells score and D-dimer testing in selected cases, achieved a negative predictive value of 99.5% (95% CI: 98–99.9%) and a sensitivity of 97.8% (95% CI: 92.4–99.7%) for ruling out DVT.

These results align with existing evidence. The latest systematic review by Zaki et al. [9] reported a pooled sensitivity of 92.3% (95% CI: 87.6–97.1%) and a negative predictive value of 97.25% (95% CI: 95.5–99.0%) for diagnosing DVT. Hercz et al. [8] found that 3-point ultrasound had a higher sensitivity than 2-point ultrasound (92% vs. 88%). The slightly higher sensitivity and negative predictive value observed in our study may be attributed to our comprehensive PUL protocol, which included compression testing every centimeter along the femoral and popliteal veins, examination of the deep femoral vein, and focused scanning of the symptomatic area. Additionally, combining PUL with risk stratification ensured follow-up ultrasounds for high-risk patients with positive D-dimer results. While this approach requires slightly more time compared to faster 2- or 3-point ultrasound methods, it is still significantly quicker and easier than whole-leg ultrasound or duplex scanning. Despite PUL being a more comprehensive test than the 2- or 3-point ultrasound, our findings indicate that PUL can be effectively implemented in a busy ED environment. Previous studies have demonstrated the high diagnostic accuracy of PUL [6–9], and our findings support its safety in routine care with potential time and resource savings. Widespread implementation in emergency departments could improve patient flow, expedite diagnosis, and reduce referrals, ultimately alleviating ED crowding and optimizing resource use.

Our study involved emergency medicine physicians and residents with varied ultrasound experience levels, demonstrating a low rate of missed DVT diagnoses. This result is likely due to effective PUL training and certification combined with adequate clinical experience to perform reliable risk stratification. While previous research is divided on whether physician experience level (resident vs. specialist) impacts diagnostic accuracy [8, 10], most would agree that increased ultrasound training improves accuracy. For instance, Turnbull et al. reported a negative predictive value (NPV) of 92% with less-experienced PUL operators, whereas Magazzini et al. achieved an NPV of 100% following intensive training [10]. Our high NPV of 99.5% indicates that a diverse group of emergency medicine physicians can safely exclude DVT using a structured, but not overly extensive, training (8 h) and certification program with 20 approved scans, which is comparable but slightly less than the 25 scans recommended in a Scandinavian guideline [14].

In this study, more than 80% of patients with suspected DVT were managed entirely in the ED without referral to a diagnostic department. This underscores the efficiency of combining PUL with risk stratification to streamline patient management, reducing the logistical burden on the ED and enhancing patient throughput. By facilitating quicker diagnoses, this approach would probably reduce patient time in the ED and reduce dependency on diagnostic departments, contributing to a more efficient healthcare delivery system. Although process times were not specifically monitored in this study, Zaki et al. demonstrated in pooled data from 4 studies that time from triage to diagnosis was significantly shorter when emergency physicians performed ultrasounds in the ED compared to reference tests conducted by radiologists [9].

This study primarily focused on the safety of ruling out DVT in the ED using PUL. We also explored bleeding prevalence among patients diagnosed with and treated for DVT in the ED. Although the study was not specifically powered for this analysis, we observed a major bleeding rate of 0.99%, with the event likely procedure-related. This finding supports the safety of initiating anticoagulation based on DVT diagnoses made with PUL in the ED. The reported specificity of PUL is 98.5% [9]. Given the observed major bleeding rate, one major bleed would occur in a patient without DVT (false positive) for every 6,734 patients examined with PUL in the ED.

### Strengths

The overall prevalence of DVT in our study was 18.4%, similar to the 23% reported by Pomero et al. [6], suggesting a representative ED population.

The study was conducted in routine care by physicians and residents with varying levels of ultrasound experience, enhancing its generalizability to the broader group of clinicians working in EDs. Furthermore, Examinations were conducted during all hours and days, supporting the generalizability of the findings to a 24/7 ED setting.

### Limitations

The 30-day follow-up revealed two false-negative diagnoses of DVT in the ED, as defined by the study criteria (diagnosis of thromboembolism within 30 days). However, it remains uncertain whether these patients had DVT at the time of their initial ED visit or whether the DVT and PE developed subsequently. If the latter were true, the results would have marginally improved but would not have altered the study’s conclusions.

It is also important to note that some cases of DVT may have been missed, with diagnoses delayed beyond 30 days, potentially leading to a lower NPV than reported. However, given that only 2 false negatives were identified out of 381 cases within 30 days, it is unlikely that the number of false negatives beyond 30 days would significantly affect the study’s conclusions. Furthermore, it is plausible that DVTs not resulting in a return visit within 30 days are small, do not progress proximally, and probably resolve spontaneously.

The follow-up relied on electronic medical records from the regional healthcare system. While thromboembolic events occurring outside the region could theoretically have been missed, this is unlikely given that most patients seek care within the region they live and would probably have been registered in outpatient records.

The study did not include non-residents, who represent a small proportion of patients with suspected DVT in our department. However, this could potentially limit the generalizability of the findings to departments with a higher proportion of non-resident patients.

Additionally, since this routine care study did not include further confirmation of DVT diagnoses, some patients diagnosed with DVT in the ED may potentially have been false positives. However, this pragmatic routine-care study builds on prior research showing a high specificity of PUL compared to reference standard tests, with reported values of up to 98.5% [6]. As a result, the number of false positives is likely small and would in any case not impact the primary results regarding the negative predictive value. To address the potential for false positives, patients with suspected DVT in areas of prior thrombosis were recommended for diagnostic department referral. The PUL training also emphasized common pitfalls, such as lymph nodes, that could be mistaken for a thrombus. Nevertheless, as false positives were not assessed in the study design, specificity could not be reported.

The primary concern with false-positive diagnoses is the risk of bleeding during anticoagulation treatment, which was typically administered for 3–6 months depending on clot location. However, the observed rate of major bleeding in this population was low, further supporting the safety of the PUL-based approach. This indicates that the conclusions regarding the safety and feasibility of the PUL routine are likely robust, even considering the risk of false positives.

## Conclusion

In this single-center study of routine emergency department (ED) care, risk stratification combined with PUL, performed by emergency medicine physicians or residents, proved to be a safe method for ruling out deep vein thrombosis (DVT). Using this approach, more than 8 of 10 patients could be diagnosed in the ED without the need for external diagnostic support.

## Data Availability

No datasets were generated or analysed during the current study.

## Abbreviations

ED: emergency department
PUL: proximal compression ultrasound
DVT: deep vein thrombosis
CDUS: complete duplex ultrasound
WLU: whole leg ultrasound
NPV: negative predictive value
EP: emergency physician
PE: pulmonary embolism
IQR: interquartile range
LMWH: low molecular weight heparin
DOAC: direct oral anticoagulant
CI: confidence interval
SD: standard deviations
POCUS: point-of-care ultrasound
